# Long-term survival rates of thalassemia patients following hematopoietic stem cell transplantation: A systematic review and meta-analysis

**DOI:** 10.1016/j.htct.2026.106490

**Published:** 2026-07-28

**Authors:** Marcella Adisuhanto, Alver Prasetya, Alius Cahyadi, Amaylia Oehadian

**Affiliations:** a Department of Internal Medicine, School of Medicine and Health Sciences, Atma Jaya Catholic University of Indonesia, Jakarta, Indonesia; b Division of Hematology and Medical Oncology, Department of Internal Medicine, Padjadjaran University, Indonesia

**Keywords:** Hematopoietic stem cell transplantation, Thalassemia, Survival rates

## Abstract

**Background:**

Hematopoietic stem cell transplantation is the sole therapeutic approach that can provide a complete cure for thalassemia. However, this procedure is associated with complications that may have life-threatening consequences. To date, long-term survival outcomes after transplantation in thalassemia have not been systematically synthesized. This study aims to specifically evaluate long-term overall survival in patients with thalassemia undergoing hematopoietic stem cell transplantation.

**Methods:**

A comprehensive search was conducted across multiple databases, including PubMed, CENTRAL, Europe PMC (incorporating medRxiv and bioRxiv), EBSCOHost (Medline), and ProQuest. The search spanned from the inception of each database through July 10, 2024, using a combination of predefined keywords: ‘Hematopoietic Stem Cell Transplantation’, ‘Thalassemia’, ‘Survival Rates’, and synonyms. Risk of bias was assessed using the Quality in Prognosis Studies (QUIPS) tool. Heterogeneity was evaluated via *I*^2^ statistics, while pooled effect estimates were calculated using the DerSimonian–Laird inverse-variance random-effects model. Statistical significance was defined as p < 0.05. Additionally, a leave-one-out sensitivity analysis was performed to ensure the robustness of the findings.

**Results:**

Out of an initial 690 records identified, four articles involving a total of 616 transplanted thalassemia patients were included in this study. The survival rates were 86.8% (95% CI: 80.1%- 92.3%) in 15 years, 89.2% (95% CI: 82.2%–96.2%) in 20 years, 82.6% (95% CI: 79.9%-85.3%) in 30 years, and 81.4% (95% CI: 74.5%–88.9%) in 39 years. The pooled survival rate was 85% (95% CI: 81%-89%; *I*^2^ = 40%). The pooled survival rate showed no significant differences in leave-one-out sensitivity analysis.

**Conclusion:**

The present meta-analysis shows relatively high long-term overall survival rates in patients with thalassemia after hematopoietic stem cell transplantation. Nonetheless, the relatively small patient population, heterogeneity in anti-thymocyte globulin implementation, and potential confounding risks intrinsic to the study necessitate cautious interpretation of the findings.

## Introduction

Thalassemia is characterized by the diminished synthesis of one of the globin polypeptide chains leading to impaired oxygen transport and chronic anemia [1,2]. The disease has a range of severity depending on the type of mutation. Overall, thalassemia leads to the development of anemia, iron overload and various complications including heart disease, cirrhosis, endocrine diseases [3]. The prevalence of thalassemia was around 2.76/100,000 in 2018 [4,5]. The prognosis of thalassemia major has shown significant improvement during the past four decades this may be explained by advanced red blood cell transfusions, prevention and management of iron overload, and new medical therapies [6]. However, conventional management through frequent blood transfusions carries significant risks, including iron overload and blood-borne infections. Consequently, hematopoietic stem cell transplantation (HSCT) has emerged as the definitive curative alternative [7,8].

Since first being performed in 1981, HSCT is the sole treatment strategy that can provide a complete cure for thalassemia [9]. HSCT is a medical technique that involves transferring healthy hematopoietic stem cells from a donor to patients who have impaired hematologic systems, such as those with thalassemia [10]. Even though HSCT is an essential treatment for thalassemia patients, it comes with several life-threatening complications and significant morbidity. HSCT can lead to several complications such as graft versus host disease (GvHD), graft failure, and serious infections. These factors result in a reduction of the long-term survival rates of individuals with thalassemia [11]. Therefore, this review intends to summarize the current knowledge regarding the survival rate of thalassemia patients who undergo HSCT.

## Methods

The Preferred Reporting Items for Systematic Reviews and Meta-Analyses (PRISMA) statement guided the conduct of this research [12]. Three independent investigators performed a detailed search for relevant studies in several databases including PubMed, Cochrane Controlled Register of Trials (CENTRAL), Europe PMC (medRxiv and bioRxiv), EBSCOHost (Medline), and ProQuest (gray literature) from the inception of each database to 10 July 2024 using keywords such as ‘Hematopoietic Stem Cell Transplantation’, ‘Thalassemia’, ‘Survival Rates’, and relevant synonyms (Supplementary Table 1).

In accordance with the PICOTS (Population, Intervention, Comparator, Outcome, Timing, Setting) approach, inclusion criteria were (P) thalassemia patients of all ages, (I) investigating hematopoietic stem cell transplantations, and (C) long term survival rates ≥10 years. There was no restriction in time and settings. Studies were excluded if the following criteria were met: (O) case reports, letter to editors, reviews, (T) non-English articles, and (S) irretrievable full-text articles.

Study selection was performed independently by three authors, with any discrepancies resolved through consultation with a fourth author. Duplicates and irrelevant articles were excluded. The authors screened the titles and abstracts obtained during the search and any work that did not satisfy the inclusion criteria was excluded. The full text of studies selected at this stage were also screened to determine their eligibility. Data extraction was performed using a collaborative online word-processing platform. Any inconsistencies were resolved through consensus-based discussion. The extracted variables encompassed author names, publication year, study design, patient demographics, and clinical outcomes.

Utilizing the Quality in Prognosis Studies tool (QUIPS), each author independently evaluated the methodological quality of included studies in order to determine the risk of bias [13,14]. Pooled effect estimates were calculated using the DerSimonian–Laird random-effects model. To account for between-study variability, an inverse-variance method with logit transformation was applied. Statistical significance was defined by a two-sided p-value <0.05, and inter-study heterogeneity was quantified using the *I*^2^ statistic. To evaluate the robustness of the pooled estimates and identify the influence of individual studies, a leave-one-out sensitivity analysis was performed, sequentially excluding each study from the meta-analysis.

## Results

**Figure 1:**
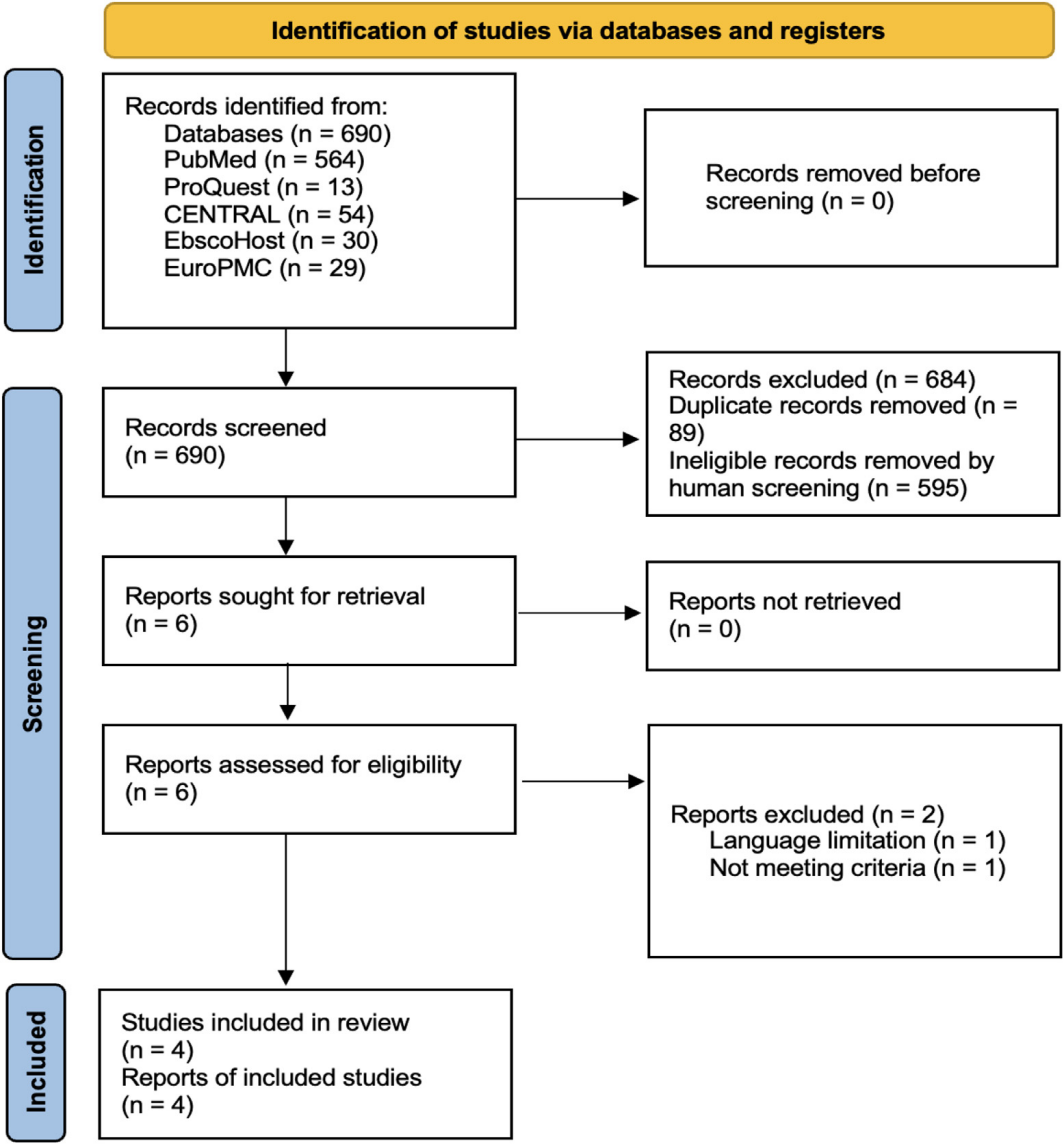
Literature search process and results.

From a total of 690 articles, 89 duplicates, and 595 ineligible records were removed. Ultimately, six studies were assessed for eligibility; of these, two were excluded because they did not meet the predefined inclusion criteria (non-English language). Four studies involving a total of 616 transplanted thalassemia patients were included in this study (Figure 1) [6,15, 16, 17].

The median follow-up duration across the studies ranged from 11–30 years. The majority of patients (n = 522) received HSCT from HLA-matched sibling donors, while the remaining 96 patients underwent transplantation from unrelated donors. The vast majority of patients (97.7%; n = 602) received bone marrow grafts, while the remainder received cord blood (n = 9), peripheral blood (n = 6), or a combination of bone marrow and cord blood (n = 1). Furthermore, 91.1% (n = 558) of the cohort was treated with busulfan-based regimens (Table 1) [6,15, 16, 17].

**Table 1:** Summary of included studies.

Author year	Type of study	patients n	Median Age at HSCT years (range)	Stem Cell Source	Regimens	HLA-typing	Median follow-up years (range)	Kaplan Meier Estimates (OS)	Risk of Bias
Caocci G. 2017 [17]	Case-control study	258	12 (1–45)	BM (n = 256); PB (n = 2)	Busulfan-based regiment (n = 208); Treosulfan-based regiment (n = 51)	HLA-identical sibling (n = 170); Unrelated donor (n = 85); HLA pheno-identical mother (n = 3)	11 (1–30)	30-year OS: 82.6% (95% CI: 79.9%−85.3%)	Low risk
Galambrun C. 2013 [15]	Retrospective observational study	108	6.2 (0.7–32)	BM (n = 96); PB (n = 3); CB (n = 8); CB plus BM (n = 1)	Busulfan-cyclophosphamide (n = 95); Fludarabine (n = 1), Busulfan-fludarabine-thiotepa (n = 7)	HLA-matched sibling donor (n = 96); phenotypic HLA-identical relative (n = 6); Matched unrelated donor (n = 6)	12 (2–21)	15-year OS: 86.8% (95% CI: 80.1%−92.3%)	Moderate risk
Santarone S. 2022 [6]	Retrospective observational study	137	10.1 (1–29)	BM (n = 135); PB (n = 1); CB (n = 1)	Busulfan-cyclophosphamide (n = 121); Busulfan-fludarabine-thiotepa (n = 12); Treosulfan-thiotepa-fludarabine (n = 4)	HLA genotypically identical sibling (n = 127); HLA phenotypically identical parent (n = 6); unrelated donor (n = 4)	30 (4–39)	39-year OS: 81.4% (95% CI: 74.5%–88.9%)	Low risk
Di Bartolomeo P. 2008 [16]	Observational study	115	9 (0.9–28)	BM (n = 115)	Busulfan-cyclophosphamide (n = 115)	HLA genotypically identical donors (n = 111); HLA phenotypically identical parent (n = 3); HLA phenotypically identical uncle (n = 1)	15 (1–24)	20-year OS: 89.2% (95% CI: 82.2%–96.2%)	Low risk

HSCT: Hematopoietic Stem Cell Transplantation; HLA: Human leukocyte antigens; OS: Overall Survival; 95% CI: 95% Confidence Interval; BM: Bone marrow; PB: Peripheral blood; CB: Cord blood.

**Figure 2:**
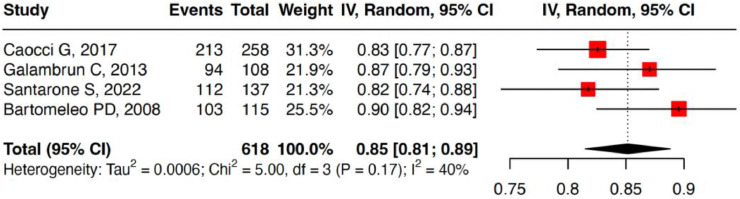
Pooled overall survival of transplanted thalassemia patients.

The pooled long-term survival rate of thalassemia patients who underwent HSCT was 85% (95% CI: 81%−89%) with *I*^2^ = 40% (Figure 2). The survival rates were 86.8% (95% CI: 80.1%–92.3%) in 15 years, 89.2% (95% CI: 82.2%–96.2%) in 20 years, 82.6% (95% CI: 79.9%–85.3%) in 30 years, and 81.4% (95% CI: 74.5%–88.9%) in 39 years [6,15, 16, 17].

**Figure 3:**
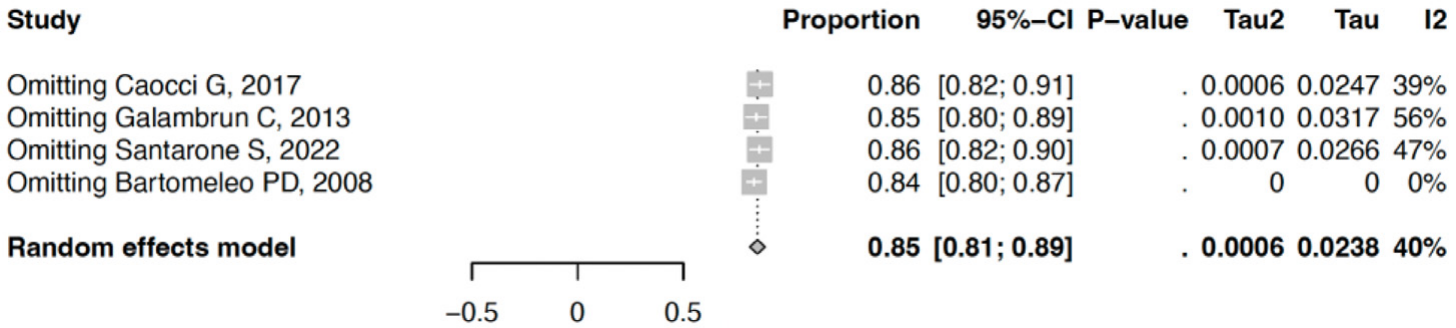
Leave-one-out sensitivity analysis.

**Figure 4:**
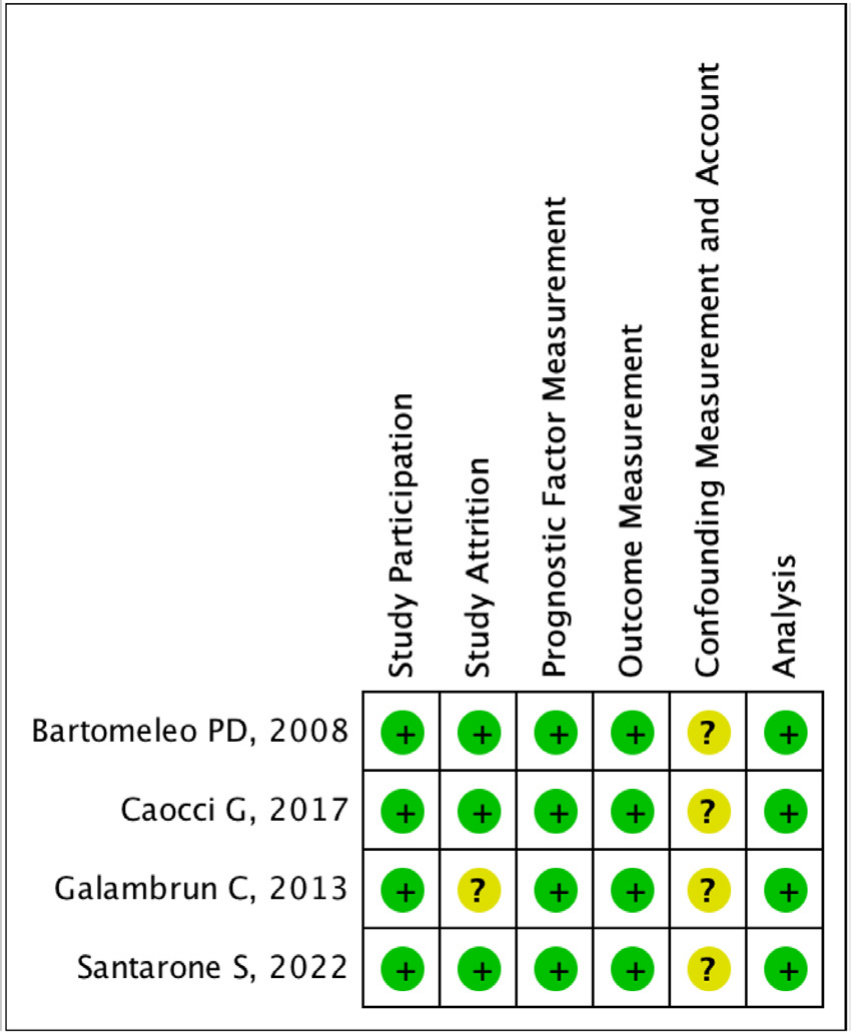
Risk of bias Summary.

Using leave-one-out sensitivity analysis, the pooled survival rate showed no significant differences when each of the studies was individually removed (Figure 3). The pooled estimates, ranging from 0.84 to 0.86, remained stable with overlapping 95% confidence intervals, therefore the results are robust and without inter-study heterogeneity. Risk of bias assessment showed that all included studies were subject to some risk in the confounding measurement and account domain, reflecting the inherent limitations of observational study designs. Overall, three studies were judged to have a low risk of bias, while one study was assessed as having a moderate risk of bias, due to additional concerns related to unreported study attrition (Figure 4). Given the potential presence of confounding factors that could influence the results, the pooled overall survival (OS) estimate may have been affected.

## Discussion

The high long-term OS rates (85%) observed in thalassemia patients post-HSCT may be attributed to the robust thalassemia-free survival (TFS) rates reported across the included studies (77.8%, 83.0%, 74.5%, and 85.7%). These favorable disease-free outcomes align with current literature and reflect significant therapeutic improvements achieved over the last two decades [18].

A study by Caocci et al. compared OS rates between HSCT and conventional therapy (CT). Survival was significantly lower in the HSCT group compared to the CT group at two years (88 ± 2% vs. 99.6 ± 0.4%), five years (87.6 ± 2.1% vs. 98 ± 0.9%), and ten years (86.4 ± 2.1% vs. 95.9 ± 1.3%; p < 0.001). Whereas, the long-term survival rates, the 30-year Kaplan-Meier probabilities of OS showed no significant difference between the groups (82.6 ± 2.7% vs. 85.3 ± 2.7%). This might be due to transplant-related mortality within the first ten years making survival in that period lower. Conversely, the higher efficacy of therapies and iron chelation contributed to an improved quality of life in the CT group [17]. However, a meta-analysis conducted by Mulas et al. found that thalassemia major patients who underwent HSCT treatment had considerably higher quality of life compared to those who received CT. The possible cause could be the elevated TFS levels in patients undergoing HSCT [19]. Another benefit of HSCT is that, according to a study by John et al., it is cost effective compared to CT [20]. Further studies are needed to confirm the comparative benefits of HSCT versus CT; such analyses are beyond the scope of the present review.

HSCT is a promising treatment for thalassemia patients to become disease free. This procedure is improving and the risks and side effects are decreasing. The adverse events that persist include infections, GvHD, secondary solid cancer, impaired gonadal function, neurologic complications, and immune thrombocytopenic purpura [6,16,21]. Allogeneic HSCT has significantly higher complications compared to autologous HSCT [22]. Recent evidence has also identified a significant correlation between allogeneic HSCT and an increased risk of cardiovascular disease [23]. In addition, a study by Gaziev et al. found that although newer protocols are associated with lower transplant-related mortality, the risk remains higher in the adult population [24]. Therefore, gene therapy could be a promising alternative treatment, as it offers the potential for disease modification while avoiding the risks associated with allogeneic transplantations [25].

This study is the only meta-analysis on long-term OS rates of thalassemia patients after HSCT. One limitation of this study was the inability to perform a subgroup analysis based on the era of transplantation (e.g., pre-1994 vs. post-1994). Galambrun et al. and other studies reported that the OS and TFS are higher in the years after 1994. Since 1994, transplant recipients have experienced improved TFS due to the introduction of anti-thymocyte globulin (ATG) for GvHD prophylaxis. The implementation of ATG significantly reduced the rejection rate from 35% to 10% [15].

The relatively small number of included studies and patients may limit the precision and generalizability of the pooled estimates and introduce potential selection bias inherent to transplanted cohorts.

## Conclusion

This meta-analysis addresses the lack of clarity regarding long-term OS in patients with thalassemia undergoing HSCT. The results indicate favorable long-term survival following HSCT, supporting its role as a curative therapeutic option for thalassemia major with an acceptable long-term risk profile. However, due to the relatively small number of patients, the potential for confounding, and differences in the implementation of ATG, the findings should be interpreted with caution.

## Data availability statement

The data that support the findings of this study are available from the corresponding author upon reasonable request.

## Conflicts of interest

No conflicts of interest

## Appendix Supplementary materials

Image, application 1
