# Supplementary material for: Long-term survival rates of thalassemia patients following hematopoietic stem cell transplantation: A systematic review and meta-analysis
Source: Hematol Transfus Cell Ther. 2026 Jul 28;48(4):106490. doi: 10.1016/j.htct.2026.106490 (PMC13448898; doi:10.1016/j.htct.2026.106490)
Supplement: Supplementary file 1 [file mmc1.docx]

**Supplementary Table 1:** Search terms

| **Database** | Search terms |
| --- | --- |
| **PubMed** | ("beta-Thalassemia"[Mesh] OR "Thalassemia"[Mesh] OR "Beta-thalassemia major"[Title/Abstract] OR "Beta Thalassemia Major"[Title/Abstract] OR "Beta Thalassemia"[Title/Abstract] OR "Thalassemia Major"[Title/Abstract] OR "Thalassemia Major"[Title/Abstract] OR "Thalassaemia Major"[Title/Abstract] OR "Thalassemia"[Title/Abstract] OR "Thalassaemia"[Title/Abstract] OR "Transfusion-Dependent Thalassemia"[Title/Abstract] OR "Transfusion-Dependent Thalassaemia"[Title/Abstract] OR "β-thalassemia"[Title/Abstract] OR "β-thalassaemia"[Title/Abstract]) AND ("Hematopoietic Stem Cell Transplantation"[MeSH Terms] OR "Bone Marrow Transplantation"[MeSH Terms] OR "Peripheral Blood Stem Cell Transplantation"[MeSH Terms] OR "Peripheral Blood Stem Cells"[MeSH Terms] OR "Cord Blood Stem Cell Transplantation"[MeSH Terms] OR "Bone Marrow Transplant"[Title/Abstract] OR "Bone Marrow Transplantation"[Title/Abstract] OR "BMT"[Title/Abstract] OR "Hematopoietic Stem Cell"[Title/Abstract] OR "Haematopoietic Stem Cell Transplantation"[Title/Abstract] OR "Hematopoietic Stem Cell Transplantation"[Title/Abstract] OR "HSCT"[Title/Abstract] OR "HCT"[Title/Abstract] OR "Peripheral Blood Cell Transplantation"[Title/Abstract] OR "Blood Cell Transplantation"[Title/Abstract] OR "PBCT"[Title/Abstract] OR "Umbilical Cord Blood"[Title/Abstract] OR "UCBT"[Title/Abstract] OR "Hematopoietic Cell Transplantation"[Title/Abstract] OR "Haematopoietic Cell Transplantation"[Title/Abstract] OR "Transplant"[Title/Abstract]) AND ("Longevity"[MeSH Terms] OR "Survival"[MeSH Terms] OR "Mortality"[MeSH Terms] OR "Disease-Free Survival"[MeSH Terms] OR "Survival Rate"[MeSH Terms] OR "Progression-Free Survival"[MeSH Terms] OR "Survival Analysis"[Mesh] OR "Kaplan-Meier Estimate"[Mesh] OR "Length of Life"[Title/Abstract] OR "Length of Life"[Title/Abstract] OR "Life Span"[Title/Abstract] OR "Overall Survival"[Title/Abstract] OR "Thalassemia-Free Survival"[Title/Abstract] OR "Thalassemia Free Survival"[Title/Abstract] OR "Thalassaemia-Free Survival"[Title/Abstract] OR "Thalassaemia Free Survival"[Title/Abstract] OR "TFS"[Title/Abstract] OR "Event Free Survival"[Title/Abstract] OR "TEFS"[Title/Abstract] OR "Survival"[Title/Abstract] OR "Disease-free survival"[Title/Abstract] OR "Disease Free Survival"[Title/Abstract] OR "Long-Term Survival"[Title/Abstract] OR "Long Term Survival"[Title/Abstract] OR "Outcomes"[Title/Abstract] OR "Outcome"[Title/Abstract] OR "Late Effects"[Title/Abstract]) |
| **ProQuest** | ti("Beta-Thalassemia" OR "Thalassemia" OR "Beta-thalassemia major" OR "Beta Thalassemia Major" OR "Beta Thalassemia" OR "Thalassemia Major" OR "Thalassaemia Major" OR "Thalassemia" OR "Thalassaemia" OR "Transfusion-Dependent Thalassemia" OR "Transfusion-Dependent Thalassaemia" OR "β-thalassemia" OR "β-thalassaemia") AND ti("Hematopoietic Stem Cell Transplantation" OR "Bone Marrow Transplantation" OR "Peripheral Blood Stem Cell Transplantation" OR "Peripheral Blood Stem Cells" OR "Cord Blood Stem Cell Transplantation" OR "Bone Marrow Transplant" OR "Bone Marrow Transplantation" OR "BMT" OR "Hematopoietic Stem Cell" OR "Haematopoietic Stem Cell Transplantation" OR "Blood Cell Transplantation" OR "PBCT" OR "Umbilical Cord Blood" OR "UCBT" OR "Hematopoietic Cell Transplantation" OR "Haematopoietic Cell Transplantation" OR "Transplant”) AND ti("Longevity" OR "Survival" OR "Mortality" OR "Disease-Free Survival" OR "Survival Rate" OR "Progression-Free Survival" OR "Survival Analysis" OR "Kaplan-Meier Estimate" OR "Length of Life" OR "Length of Life" OR "Life Span" OR "Overall Survival" OR "Thalassemia-Free Survival" OR "Thalassemia Free Survival" OR "Thalassaemia-Free Survival" OR "Thalassaemia Free Survival" OR "TFS" OR "Event Free Survival" OR "TEFS" OR "Disease-free survival" OR "Disease Free Survival" OR "Long-Term Survival" OR "Long Term Survival" OR "Outcomes" OR "Outcome" OR "Late Effects") |
| **CENTRAL** | "beta-Thalassemia" OR "Thalassemia" OR "Beta-thalassemia major" OR "Beta Thalassemia Major" OR "Beta Thalassemia" OR "Thalassemia Major" OR "Thalassemia Major" OR "Thalassaemia Major" OR "Thalassemia" OR "Thalassaemia" OR "Transfusion-Dependent Thalassemia" OR "Transfusion-Dependent Thalassaemia" OR "β-thalassemia" OR "β-thalassaemia" in Title Abstract Keyword AND "Hematopoietic Stem Cell Transplantation" OR "Bone Marrow Transplantation" OR "Peripheral Blood Stem Cell Transplantation" OR "Peripheral Blood Stem Cells" OR "Cord Blood Stem Cell Transplantation" OR "Bone Marrow Transplant" OR "Bone Marrow Transplantation" OR "BMT" OR "Hematopoietic Stem Cell" OR "Haematopoietic Stem Cell Transplantation" OR "Hematopoietic Stem Cell Transplantation" OR "HSCT" OR "HCT" OR "Peripheral Blood Cell Transplantation" OR "Blood Cell Transplantation" OR "PBCT" OR "Umbilical Cord Blood" OR "UCBT" OR "Hematopoietic Cell Transplantation" OR "Haematopoietic Cell Transplantation" OR "Transplant" in Title Abstract Keyword AND "Longevity" OR "Survival" OR "Mortality" OR "Disease-Free Survival" OR "Survival Rate" OR "Progression-Free Survival" OR "Survival Analysis" OR "Kaplan-Meier Estimate" OR "Length of Life" OR "Length of Life" OR "Life Span" OR "Overall Survival" OR "Thalassemia-Free Survival" OR "Thalassemia Free Survival" OR "Thalassaemia-Free Survival" OR "Thalassaemia Free Survival" OR "TFS" OR "Event Free Survival" OR "TEFS" OR "Survival" OR "Disease-free survival" OR "Disease Free Survival" OR "Long-Term Survival" OR "Long Term Survival" OR "Outcomes" OR "Outcome" OR "Late Effects" in Title Abstract |
| **EbscoHOST** | TI ("Beta-Thalassemia" OR "Thalassemia" OR "Beta-thalassemia major" OR "Beta Thalassemia Major" OR "Beta Thalassemia" OR "Thalassemia Major" OR "Thalassaemia Major" OR "Thalassemia" OR "Thalassaemia" OR "Transfusion-Dependent Thalassemia" OR "Transfusion-Dependent Thalassaemia" OR "β-thalassemia" OR "β-thalassaemia") AND TI ("Hematopoietic Stem Cell Transplantation" OR "Bone Marrow Transplantation" OR "Peripheral Blood Stem Cell Transplantation" OR "Peripheral Blood Stem Cells" OR "Cord Blood Stem Cell Transplantation" OR "Bone Marrow Transplant" OR "Bone Marrow Transplantation" OR "BMT" OR "Hematopoietic Stem Cell" OR "Haematopoietic Stem Cell Transplantation" OR "Blood Cell Transplantation" OR "PBCT" OR "Umbilical Cord Blood" OR "UCBT" OR "Hematopoietic Cell Transplantation" OR "Haematopoietic Cell Transplantation" OR "Transplant”) AND TI ("Longevity" OR "Survival" OR "Mortality" OR "Disease-Free Survival" OR "Survival Rate" OR "Progression-Free Survival" OR "Survival Analysis" OR "Kaplan-Meier Estimate" OR "Length of Life" OR "Length of Life" OR "Life Span" OR "Overall Survival" OR "Thalassemia-Free Survival" OR "Thalassemia Free Survival" OR "Thalassaemia-Free Survival" OR "Thalassaemia Free Survival" OR "TFS" OR "Event Free Survival" OR "TEFS" OR "Disease-free survival" OR "Disease Free Survival" OR "Long-Term Survival" OR "Long Term Survival" OR "Outcomes" OR "Outcome" OR "Late Effects") |
| **EuroPMC** | (TITLE:"Beta-Thalassemia" OR TITLE:"Thalassemia" OR TITLE:"Beta-thalassemia major" OR TITLE:"Beta Thalassemia Major" OR TITLE:"Beta Thalassemia" OR TITLE:"Thalassemia Major" OR TITLE:"Thalassaemia Major" OR TITLE:"Thalassemia" OR TITLE:"Thalassaemia" OR TITLE:"Transfusion-Dependent Thalassemia" OR TITLE:"Transfusion-Dependent Thalassaemia" OR TITLE:"β-thalassemia" OR TITLE:"β-thalassaemia") AND (TITLE:"Hematopoietic Stem Cell Transplantation" OR TITLE:"Bone Marrow Transplantation" OR TITLE:"Peripheral Blood Stem Cell Transplantation" OR TITLE:"Peripheral Blood Stem Cells" OR TITLE:"Cord Blood Stem Cell Transplantation" OR TITLE:"Bone Marrow Transplant" OR TITLE:"Bone Marrow Transplantation" OR TITLE:"Hematopoietic Stem Cell" OR TITLE:"Haematopoietic Stem Cell Transplantation" OR TITLE:"Blood Cell Transplantation" OR TITLE:"Umbilical Cord Blood" OR TITLE:"Hematopoietic Cell Transplantation" OR TITLE:"Haematopoietic Cell Transplantation" OR TITLE:"Transplant”) AND (TITLE:"Longevity" OR TITLE:"Survival" OR TITLE:"Mortality" OR TITLE:"Disease-Free Survival" OR TITLE:"Survival Rate" OR TITLE:"Progression-Free Survival" OR TITLE:"Survival Analysis" OR TITLE:"Kaplan-Meier Estimate" OR TITLE:"Length of Life" OR TITLE:"Length of Life" OR TITLE:"Life Span" OR TITLE:"Overall Survival" OR TITLE:"Thalassemia-Free Survival" OR TITLE:"Thalassemia Free Survival" OR TITLE:"Thalassaemia-Free Survival" OR TITLE:"Thalassaemia Free Survival" OR TITLE:"Event Free Survival" OR TITLE:"Disease-free survival" OR TITLE:"Disease Free Survival" OR TITLE:"Long-Term Survival" OR TITLE:"Long Term Survival" OR TITLE:"Outcomes" OR TITLE:"Outcome" OR TITLE:"Late Effects") |
